# Manganese Carbonyl Complexes as Selective Electrocatalysts for CO_2_ Reduction in Water and Organic Solvents

**DOI:** 10.1021/acs.accounts.1c00692

**Published:** 2022-03-14

**Authors:** Bhavin Siritanaratkul, Catherine Eagle, Alexander J. Cowan

**Affiliations:** Stephenson Institute for Renewable Energy and the Department of Chemistry, University of Liverpool, Liverpool L69 7ZF, U.K.

## Abstract

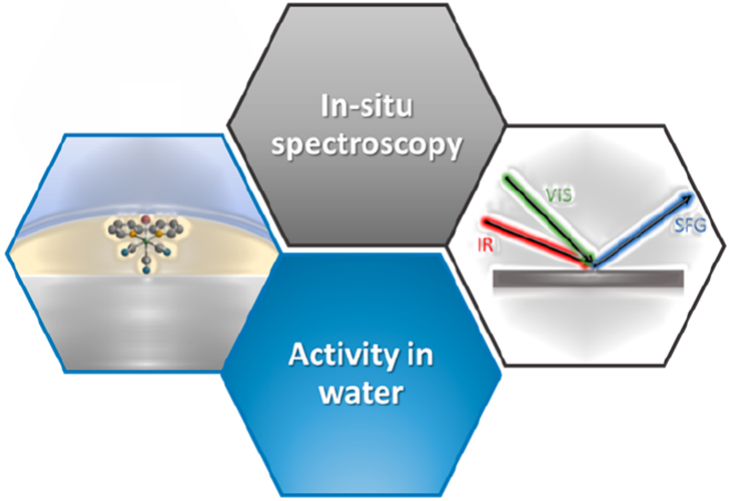

The electrochemical reduction of CO_2_ provides a way to sustainably generate carbon-based fuels and feedstocks. Molecular CO_2_ reduction electrocatalysts provide tunable reaction centers offering an approach to control the selectivity of catalysis. Manganese carbonyl complexes, based on [Mn(bpy)(CO)_3_Br] and its derivatives (bpy = 2,2′-bipyridine), are particularly interesting due to their ease of synthesis and the use of a first-row earth-abundant transition metal. [Mn(bpy)(CO)_3_Br] was first shown to be an active and selective catalyst for reducing CO_2_ to CO in organic solvents in 2011. Since then, manganese carbonyl catalysts have been widely studied with numerous reports of their use as electrocatalysts and photocatalysts and studies of their mechanism.

This class of Mn catalysts only shows CO_2_ reduction activity with the addition of weak Brønsted acids. Perhaps surprisingly, early reports showed increased turnover frequencies as the acid strength is increased without a loss in selectivity toward CO evolution. It may have been expected that the competing hydrogen evolution reaction could have led to lower selectivity. Inspired by these works we began to explore if the catalyst would work in protic solvents, namely, water, and to explore the pH range over which it can operate. Here we describe the early studies from our laboratory that first demonstrated the use of manganese carbonyl complexes in water and then go on to discuss wider developments on the use of these catalysts in water, highlighting their potential as catalysts for use in aqueous CO_2_ electrolyzers.

Key to the excellent selectivity of these catalysts in the presence of Brønsted acids is a proton-assisted CO_2_ binding mechanism, where for the acids widely studied, lower p*K*_a_ values actually favor CO_2_ binding over Mn–H formation, a precursor to H_2_ evolution. Here we discuss the wider literature before focusing on our own contributions in validating this previously proposed mechanism through the use of vibrational sum frequency generation (VSFG) spectroelectrochemistry. This allowed us to study [Mn(bpy)(CO)_3_Br] while it is at, or near, the electrode surface, which provided a way to identify new catalytic intermediates and also confirm that proton-assisted CO_2_ binding operates in both the “dimer” and primary (via [Mn(bpy)(CO)_3_]^−^) pathways. Understanding the mechanism of how these highly selective catalysts operate is important as we propose that the Mn complexes will be valuable models to guide the development of new proton/acid tolerant CO_2_ reduction catalysts.

## Key References

- WalshJ. J.; NeriG.; SmithC. L.; CowanA. J.Electrocatalytic CO2 reduction with a membrane supported manganese catalyst in aqueous solution. Chem. Commun.2014, 50, 12698–1270110.1039/c4cc06404f25204759.^1^*This work used a simple approach to immobilize the Mn complex on a carbon support allowing for its study in aqueous solvent for the first time, demonstrating that CO_2_ reduction selectivity was retained*.
- WalshJ. J.; NeriG.; SmithC. L.; CowanA. J.Water-Soluble Manganese Complex for Selective Electrocatalytic CO2 Reduction to CO. Organometallics2019, 38, 1224–1229.^2^*Here we showed the activity and selectivity of a carboxylic acid derivative in water across a wide pH range*.
- NeriG.; WalshJ. J.; TeobaldiG.; DonaldsonP. M.; CowanA. J.Detection of catalytic intermediates at an electrode surface during carbon dioxide reduction by an earth-abundant catalyst. Nature Catalysis2018, 1, 952–959.^3^*This study used vibrational sum-frequency generation spectroscopy to follow the reaction mechanisms of the Mn catalyst transiently at an electrode during carbon dioxide reduction*.
- NeriG.; DonaldsonP. M.; CowanA. J.In situ study of the low overpotential “dimer pathway” for electrocatalytic carbon dioxide reduction by manganese carbonyl complexes. Phys. Chem. Chem. Phys., 2019, 21, 7389–73973090693810.1039/c9cp00504h.^4^*Here we examined the surface behavior of the Mn catalyst as it arrives at the electrode and also explored the mechanism of the less studied lower overpotential reaction pathway*.

## 1 Introduction

Electrochemical CO_2_ reduction will be needed to enable a circular carbon economy and it is proposed to play an important role in managing CO_2_ emissions.^5−7^ Electrochemical CO_2_ conversion at scale is expected to make use of point sources of CO_2_, such as flue gas from heavy industries. Metal electrodes and metallic electrocatalysts^8^ deposited onto high surface area supports have demonstrated that reduction of pure CO_2_ feeds can achieve high current densities (up to 1 A cm^–2^) in CO_2_ electrolyzers.^9^ However, as the CO_2_ concentration is decreased and impurities such as O_2_, NO_*x*_, and SO_*x*_, are added to simulate a typical flue gas stream, changes in selectivity have been reported.^10,11^ Molecular catalysts^12−15^ provide an opportunity to achieve desired reactant and product selectivity by altering the ligands surrounding the metal center to tune the reaction center’s electronics and steric bulk. Therefore, they are particularly interesting as both models of how CO_2_ selectivity can be controlled and potential practical-scale catalysts for application in an immobilized configuration.

**Figure 1:**
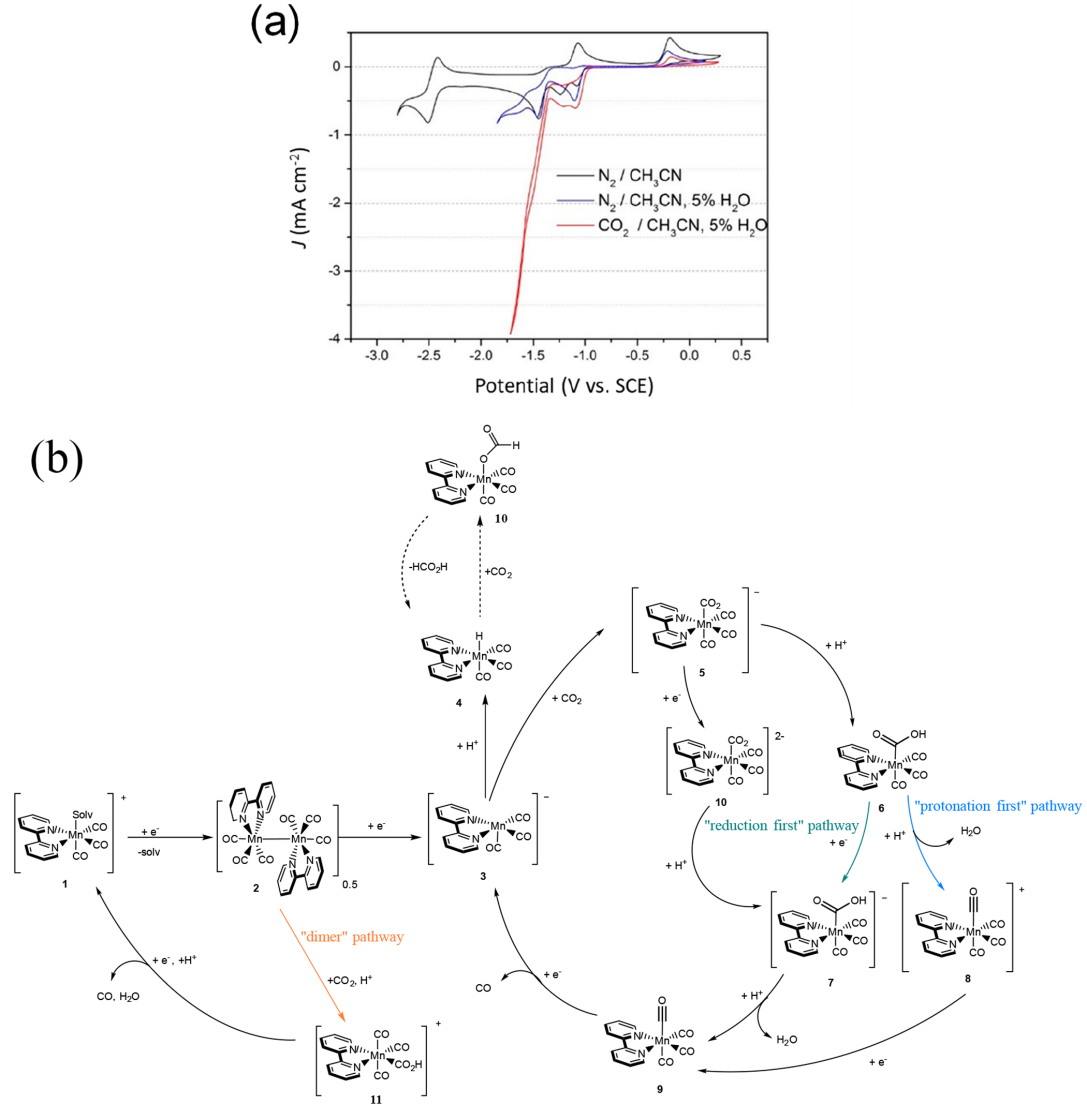
(a) CV of [Mn(bpy)(CO)_3_Br] in CH_3_CN at a glassy carbon electrode under the conditions indicated. (b) Proposed catalytic cycle of [Mn(bpy)(CO)_3_Br]. Three main pathways for CO_2_ reduction to CO have been evidenced through a wide range of spectroscopic, electrochemical, and theoretical studies.^22^ These are the dimer pathway (orange), protonation first pathway (blue), and reduction first pathway (green). An additional proposed pathway to form formate or formic acid is also shown^28^ (dashed lines). Panel b adapted from ref (3) with permission from Springer Nature.

A widely studied class of molecular electrocatalysts is those based on [*fac-*Re(bpy)(CO)_3_Cl] (bpy = 2,2′-bipyridine, hereafter the *fac-* is assumed for all tricarbonyl structures unless otherwise stated). This catalyst was first reported in the 1980s to produce CO both photocatalytically and electrochemically from CO_2_, displaying high Faradaic efficiencies for CO and good stability under electrocatalytic conditions.^16,17^ Despite these promising results, Re has low natural abundance.^18^ Early on, [Mn(bpy)(CO)_3_Br] was examined as a possible alternative high-abundance catalyst, but initial reports in organic solvents noted a lack of activity toward CO_2_.^19^ It was not until 2011 in a breakthrough study by Deronzier and co-workers^20^ that [Mn(bpy-R)(CO)_3_Br] (R = H or alkyl group at the 4,4′ position) was shown to be an active electrocatalyst for CO_2_ reduction in organic solvents, but only when a Brønsted acid was added. A typical cyclic voltammogram of [Mn(bpy)(CO)_3_Br] in acetonitrile, similar to that measured in those first reports, is shown in Figure 1a, and a proposed catalytic cycle is shown in Figure 1b. Under N_2_, initial reduction at −1.2 V_SCE_ results in the loss of Br^–^ and then dimerization to form [Mn(bpy)(CO)_3_]_2_. This dimer complex is reduced at −1.5 V_SCE_ to form the main catalytically active species [Mn(bpy)(CO)_3_]^−^ as indicated by the large increase in current under CO_2_ and in the presence of a proton source. In competition with CO evolution is H_2_ production, which occurs via the formation of [Mn(bpy)(CO)_3_H].

The initial study by Deronzier and colleagues^20^ led to a large number of follow-on works on this class of catalyst, of which several reviews exist.^13,21−24^ In this Account, we discuss one of the most interesting aspects, the need for a Brønsted acid for any measurable CO_2_ reduction to occur.^16,17^ This finding was confirmed in a study by the Kubiak group in 2013, on a derivative, [Mn(bpy(^t^Bu)_2_)(CO)_3_Br], in acetonitrile with the addition of the weak acids water, methanol, and 2,2,2-trifluoroethanol (TFE), where higher turnover frequencies were achieved than with the parent complex.^25^ Also, the turnover frequency of the Mn catalyst was shown to increase with acid strength, and in general with higher concentrations of acid, without a loss in selectivity toward CO_2_ reduction. These experiments on the complex in aprotic solvents with an acid source led us to ask in 2014,^1^ could the performance of [Mn(bpy)(CO)_3_Br] be further improved by use in a protic solvent, in particular water?

Developing CO_2_ reduction catalysts that are selective in water is important; in a practical electrolyzer, the CO_2_ reduction reaction will need to be coupled to a sustainable oxidation reaction, presumably water oxidation. In particular, there is current interest in understanding how to develop systems that can selectively reduce CO_2_ in an acidic environment^26^ as operating CO_2_ electrolyzers at high pH leads to carbonate formation with consequential decreased CO_2_ conversion efficiencies.^27^ With conventional metal catalysts (e.g., Ag, Au, or Cu) operating at low pH is challenging due to competitive hydrogen evolution as a result of the high proton concentration. Therefore, the development and mechanistic study of molecular electrocatalysts that show high selectivity to CO_2_ reduction in the presence of high proton concentrations is of great interest to the field. Here we describe in section 2 the use of this class of Mn catalysts for CO_2_ reduction in water, focusing on early work from our own laboratory before discussing wider developments in the field. In section 3, we discuss mechanistic studies on the role of the acid source in the CO_2_ reduction mechanism in an effort to understand how these catalysts achieve selectivity. In particular, we introduce the use of vibrational sum frequency generation (VSFG) spectroscopy, which confirmed a previously proposed proton-assisted CO_2_ binding mechanism for the main catalytic pathways, rationalizing why these catalysts can operate even in proton-rich environments.

## 2 [Mn(bpy)(CO)_3_Br] and Its Derivatives as CO_2_ Reduction Catalysts in Water

To test how [Mn(bpy)(CO)_3_Br] behaved in the presence of aqueous electrolytes, we initially applied a simple approach previously described for a range of catalysts including [Re(bpy)(CO)_3_Br],^29^ where we deposited the Mn complex directly onto a glassy carbon electrode (GCE) using a Nafion ionomer support.^1^ Direct study of the mechanism of the catalyst within the Nafion membrane is challenging but CVs indicated that despite being immobilized and used at pH 7, the catalyst showed very similar behavior to that observed when it is dissolved in aprotic solvents with the largest current enhancement under CO_2_ occurring following the formation of [Mn(bpy)(CO)_3_]^−^. Intriguingly in the Nafion membrane, dimerization is believed to still occur following initial reduction of [Mn(bpy)(CO)_3_Br], prior to the formation of the active [Mn(bpy)(CO)_3_]^−^ catalyst, as indicated by the oxidation peak of [Mn_2_(bpy)_2_(CO)_6_]. A linear dependence of peak current with scan rate for the reduction of [Mn(bpy)(CO)_3_Br] indicated that the complex was not solubilized in the polymer, and no evidence of Mn loss into the electrolyte was found suggesting that dimer formation was the result of electroactive aggregates; however definitive evidence of the mechanism of dimerization within Nafion is still missing. Regardless of the possible mechanisms of dimerization the most important outcome of this first study was that once formed [Mn(bpy)(CO)_3_]^−^ displayed good selectivity for CO_2_ reduction in water at pH 7 with a CO/H_2_ ratio of 2:1 being achieved at −1.4 V_Ag/AgCl_ and TON of up to 470. This demonstrated the viability of using this complex in protic solvents and indicates that CO_2_ reduction selectivities on par with those seen in aprotic solvents could be achieved.

**Figure 2:**
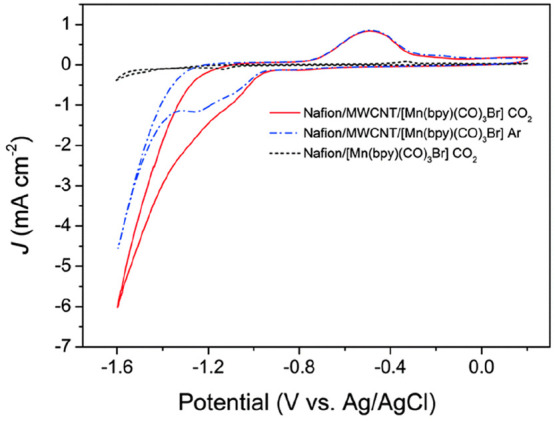
Cyclic voltammetry of [Mn(bpy)(CO)_3_Br] immobilized in a Nafion film on a glassy carbon electrode in 30 mM phosphate buffer, pH ∼7, showing the current enhancement from MWCNT addition. Reproduced from ref (1) with permission from the Royal Society of Chemistry.

In the first studies of GCE/[Mn(bpy)(CO)_3_Br]/Nafion electrodes, current densities were low (0.3 mA cm^–2^) due to the majority of the catalyst present being electro-inactive. The addition of multiwalled carbon nanotubes (MWCNTs), increasing the electroactive content, led to a large increase in current density under CO_2_ (up to 3 mA cm^–2^, Figure 2), albeit with a partial loss in CO/H_2_ selectivity (dropping to ∼1:2).^1^ A subsequent study investigated a wider range of Mn complexes that contained modifications to the 4,4′ positions of the 2,2′-bipyridine ligand immobilized in a similar manner with MWCNTs.^30^ Among the complexes studied [Mn(bpy(^t^Bu)_2_)(CO)_3_Br], which was first reported by Kubiak and colleagues,^25^ gave the highest level of selectivity toward CO (CO/H_2_ ≈ 1); however the CO partial current density was lower than the original unmodified bipyridine complex. Also studied were [Mn(bpy(OH)_2_)(CO)_3_Br] and [Mn(bpy(COOH)_2_)(CO)_3_Br], but both complexes gave disappointing levels of selectivity when immobilized, with CO/H_2_ ≈ 0.28 and 0.33, respectively.^30^ The initial studies of the Mn catalysts deposited on GCE made use of the low solubility of [Mn(bpy)(CO)_3_Br] in water;^1,30^ however relatively low electroactive contents were achieved, and the current densities reported above are ∼30 times lower than is required for application in a practical electrolyzer (>100 mA cm^–2^).

Subsequent studies have reported more advanced approaches to immobilize manganese carbonyl catalysts with several achieving notably higher current densities. Reisner and colleagues developed a derivative where the catalyst was anchored to carbon nanotubes through a pyrene-modified bipyridine ligand, which was found to show a stable current density of 0.5 mA cm^–2^ (−1.1 V_SHE_) with a good selectivity for CO production (maximum Faradaic efficiency of 34%) at pH 7.4.^28^ Interestingly this system also produced appreciable concentrations of formate, which is not a common observation in other electrocatalytic studies in water described below. Excellent selectivity for CO production (>80% Faradaic efficiency) and a stable current density of 5 mA cm^–2^ were reported from a polymerized manganese carbonyl complex on MWCNTs in a pH 7 electrolyte when K^+^ ions were present at high concentrations.^31^ Of great relevance is also the work of Vizza and co-workers who prepared [Mn(apbpy)(CO)_3_Br] (apbpy = 4-(4-aminophenyl)-2,2′-bipyridine), which can be electrochemically grafted onto carbon cloth.^32^ In this way, electrodes with the catalytic center covalently bound onto the support can be prepared and achieved Faradaic efficiencies of ∼60% for CO production at −1.4 V_Ag/AgCl_ in CO_2_ saturated KHCO_3_. Very recently, studies of this catalyst bound onto a gas diffusion electrode showed that the mass normalized turnover frequencies of the catalyst exceeded those of a benchmark Au catalyst.^33^ Another strategy to achieve higher electroactive concentrations of catalytic centers, and therefore potentially higher current densities, is to incorporate the Mn catalytic center directly within a high surface area porous framework. Examples that use a Mn catalytic center for CO_2_ reduction in water include a conjugated microporous polymer^34^ and a covalent organic framework^35^ with the latter achieving an impressive CO partial current density of 11 mA cm^–2^ and selectivity (55% Faradaic efficiency) at pH 7.4.^33^

**Figure 3:**
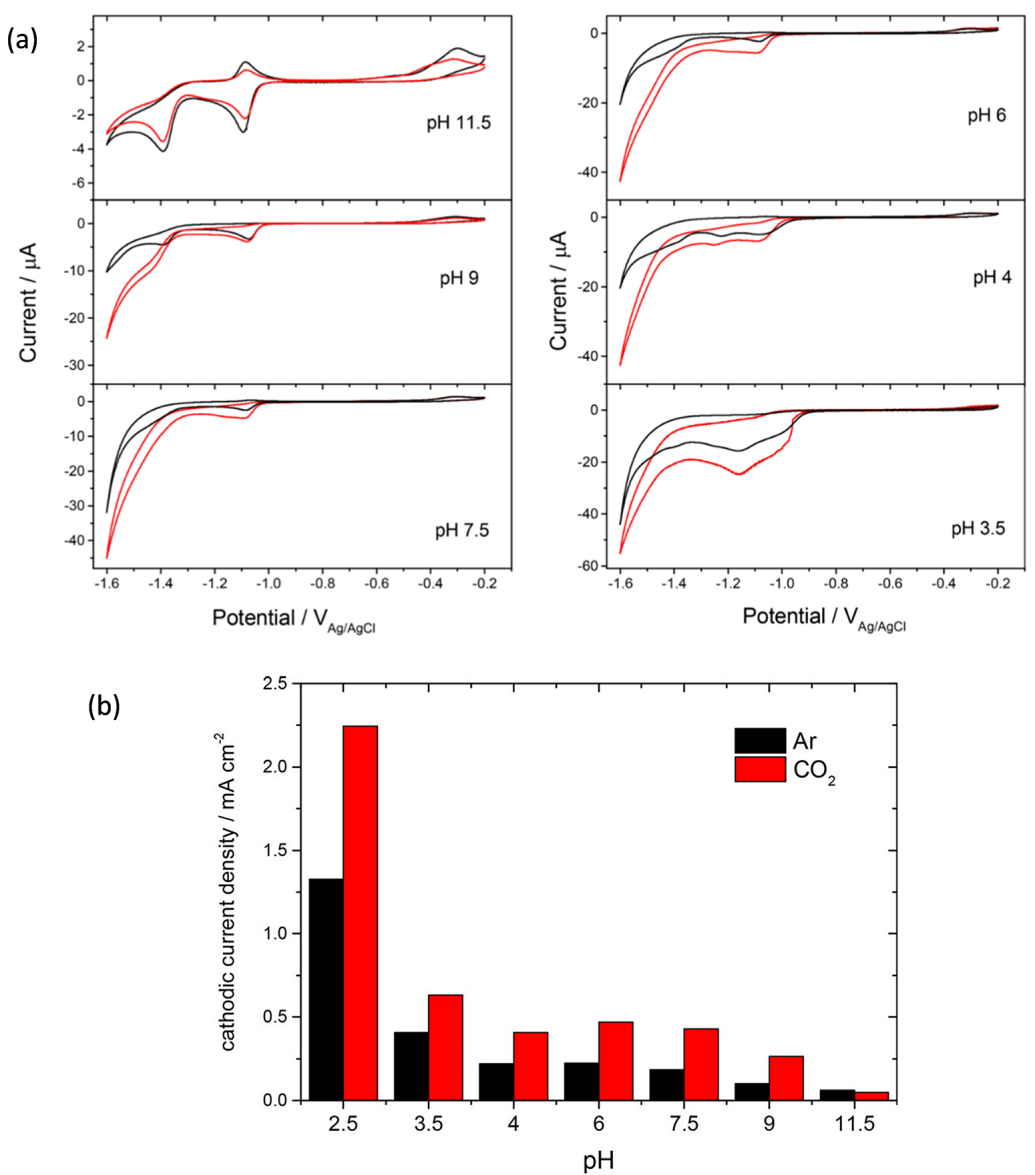
[Mn^I^(bpy)(COOH)_2_)(CO)_3_Br] is a water-soluble CO_2_ reduction catalyst that shows good selectivity toward CO production at pH 9. CVs are shown at a range of pH values under Ar (black) and under CO_2_ (red), recorded at 100 mV s^–1^ (a), current at −1.5 V_Ag/AgCl_ vs pH under argon (black) and CO_2_ (red) (b). Reproduced with permission from ref (2). Copyright 2019 American Chemical Society.

The examples of [Mn(bpy)(CO)_3_Br] derivatives immobilized onto and into electrode supports for CO_2_ reduction in water have demonstrated that a good level of selectivity (typically ≥1:1 CO/H_2_) can be achieved at pH ∼7. However, it is difficult to quantify the intrinsic selectivity of the catalyst due to the possibility of hydrogen being evolved from the carbon support or impurities within. In order to better understand the behavior of the Mn catalyst in a wide pH range, we also developed a water-soluble Mn diimine CO_2_ reduction complex [Mn(bpy(COOH)_2_)(CO)_3_Br], where bpy(COOH)_2_ = 4,4′-dicarboxy-2,2′-bipyridine. The solubility of the catalyst allowed for experiments using a Hg amalgam electrode, which has a high overpotential for hydrogen evolution making it ideal for analytical electrochemistry in water at a range of pH values.^2^ UV/vis spectroscopy showed a pH dependence due to the changing protonation state of the carboxylate groups and also indicated that the bromide ligand was readily displaced by water at open circuit. In contrast to the equivalent Re complex^36^ where the displacement of the aqua ligand by bicarbonate led to a loss in solubility at some pH values under CO_2_, the Mn analogue retained solubility under CO_2_, and no evidence of carbonate/bicarbonate ligation was observed.^12^ CVs of [Mn^I^(bpy(COO)_2_)(CO)_3_]^−^ under Ar and CO_2_ at a range of pH values were similar to other complexes from this class in conventional organic solvents with an initial reduction between −1.0 and −1.1 V_Ag/AgCl_, depending upon pH, leading to loss of a solvent ligand and dimer formation, and a further reduction between −1.4 and −1.3 V_Ag/AgCl_ formed the active [Mn(bpy(COO)_2_)(CO)_3_]^3–^ (Figure 3a). At the highest pH values studied (>9), minimal CO_2_ reduction occurs, presumably due to a combination of low concentrations of available CO_2_ and H^+^. Bulk electrolysis was only carried out at a single pH in this initial communication but notably between pH 9 and 2.5 a large enhancement in catalytic current occurred under CO_2_ with the greatest increases in current under CO_2_ occurring at the lowest pH values (Figure 3b). This indication of CO_2_ catalysis even at the lowest pH values studied is of particular interest because at pH < 4 bicarbonate formation is no longer a significant process; therefore the development and study of catalysts that can operate selectively toward CO_2_ reduction under these conditions may provide a way to enable acid CO_2_ electrolyzers.^26^

## 3 Mechanistic Studies on [Mn(bpy)(CO)_3_Br] at Electrode Surfaces: Understanding the Role of the BrØnsted Acid to Rationalize the Selectivity toward CO_2_

The electrochemical behavior of [Mn(bpy)(CO)_3_Br] has been studied in detail using a wide range of spectroscopic techniques. Covering these in detail is not the aim of this Account, and a more comprehensive review is provided by Grills et al.^22^ Instead we focus on attempts to understand how these Mn catalysts retain selectivity for CO_2_ reduction to CO in protic environments. As noted in section 1, two key studies were the original work of Deronzier and colleagues who noted the need for an acid source for catalysis to occur^20^ and work from the Kubiak group on the behavior of [Mn(bpy(^t^Bu)_2_)(CO)_3_Br] with a range of acids.^25^ In Kubiak’s 2013 study, it was proposed that the Brønsted acid was required to protonate the initially formed CO_2_ adduct, with protonation either stabilizing the Mn–CO_2_ species or facilitating the cleavage of a C–O bond. DFT calculations following on from this work by Carter and colleagues confirmed that without an acid present CO_2_ binding was endergonic.^37,38^ But once phenol was added, the process became exergonic, and initial CO_2_ binding was followed by the barrierless, strongly exergonic protonation of the Mn–CO_2_ adduct.^37,38^ This finding has since been further validated and expanded upon in DFT calculations where TFE is the acid source, which showed a dual role for the acid, stabilizing the Mn–CO_2_ adduct with subsequent rapid protonation and exergonic carbonation of the conjugate base providing additional driving force for the overall generation of a Mn–CO_2_H intermediate.^39^ The DFT studies of the Carter group also represented the first report of the presence of two catalytic pathways for CO evolution following proton assisted CO_2_ binding to [Mn(bpy)(CO)_3_]^−^, labeled the “protonation first” and “reduction first” pathways in Figure 1b. These calculations provided a rationale for the excellent selectivity and improved turnover frequency in the presence of stronger acids, and a framework by which we can understand the selectivity of the catalysts in water. However, direct detection of many of the short-lived intermediates proposed has historically been a challenge with conventional spectroscopies where the need to electrochemically generate high concentrations of species in the bulk inevitably makes the detection of short-lived transient species at the electrode surface difficult.

**Figure 4:**
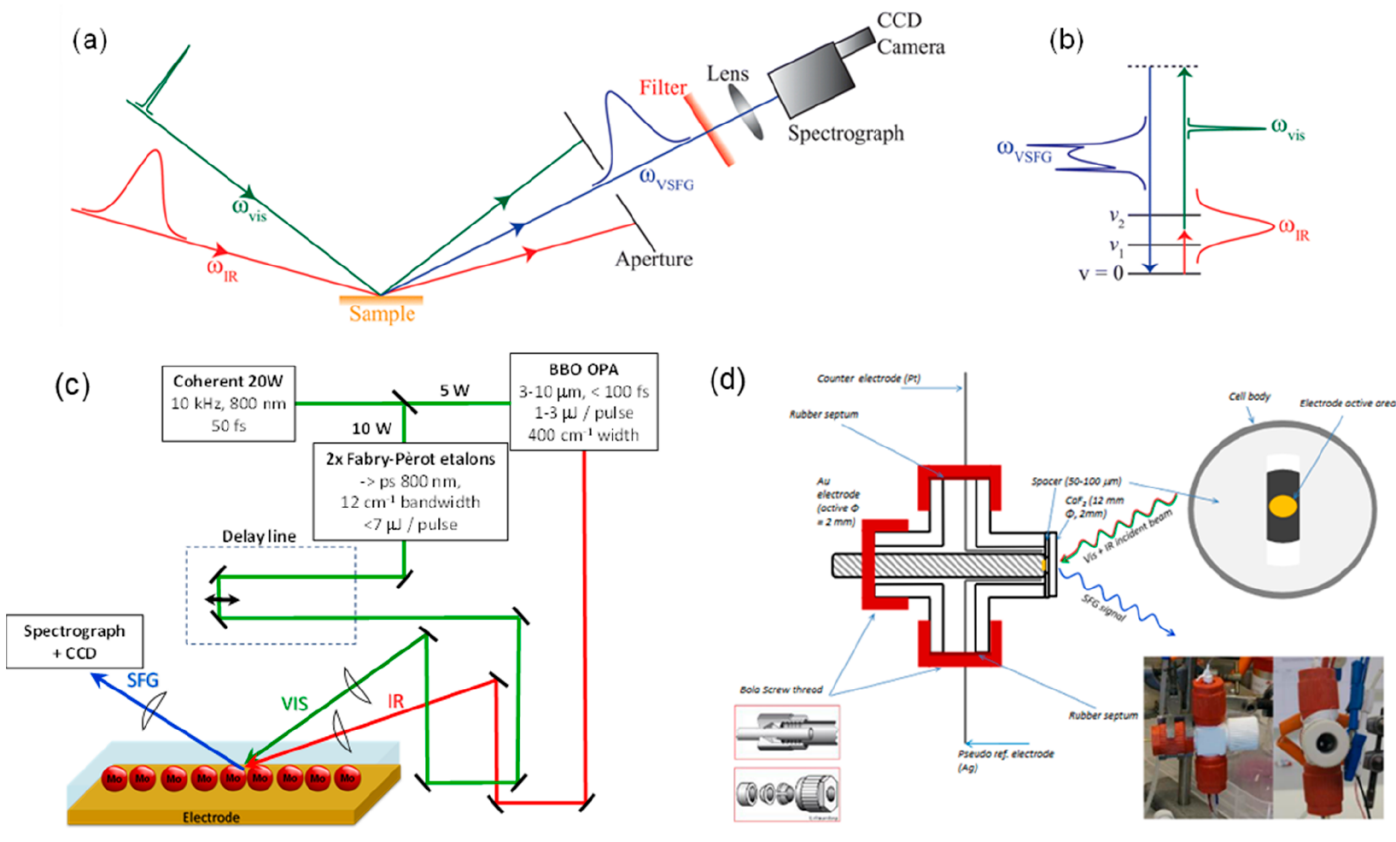
(a,b) Broad-band (<150 fs) mIR VSFG experiments. The data shown here uses a broad-band setup where a broad-band mIR pulse is incident on the electrode surface and overlapped with a narrow-band (>1 ps) visible laser pulse. (c) Experimental setup used in the VSFG data in refs (3, 4, and 40) at the UK Central Laser Facility and (d) SEC cell for VSFG with thin path-length. Panels a and b reproduced from ref (41) with permission from the PCCP Owner Societies. Panel d reproduced with permission from ref (40). Copyright 2017 American Chemical Society.

Our own contribution has focused on using vibrational sum frequency generation (VSFG) spectroscopy to study Mn catalysts at the electrode surface in the presence of a range of acids, with the aim of validating the calculated role of the Brønsted acid in CO_2_ reduction. In a VSFG experiment, two incident, short laser pulses are overlapped on the sample interface (in this case the working electrode surface), and the light is generated at the sum of the frequency of the two input pulses (Figure 4a,b). In our experiments, we use a broad-band (typically 500 cm^–1^ fwhm, 50 fs) tunable mid-infrared (mIR) laser and a fixed wavelength visible (800 nm) laser that has a picosecond pulse duration and a time asymmetric shape. Both of these are transmitted through a thin-layer of electrolyte to the electrode surface (Figure 4c,d; for full details of the experimental apparatus, see ref (40)). When the mIR laser frequency is resonant with a sum-frequency active vibrational mode, the VSFG light intensity is significantly increased and a vibrational spectrum of the species can be recorded. VSFG is often described as surface selective because signals are only generated in a non-centrosymmetric environment under the electric dipole approximation.^41,42^ However, it is important to note that across the electrical double layer structure ordering can occur giving rise to VSFG signals and contributions from third order nonlinear polarization terms can also arise from molecules throughout the double layer; therefore this statement is not strictly correct.^43,44^ Nonetheless VSFG spectroscopy provides a powerful way to study molecular electrocatalysts while they are near (within the double layer structure) or at the electrode surface, as sufficient ordering can occur due to the large electric fields present and the use of catalysts with nonzero dipole moments. A detailed review on the application of the technique to molecular electrocatalysts, which describes in more detail the experimental considerations and the route by which spectra are assigned and fitted, is available.^41^

**Figure 5:**
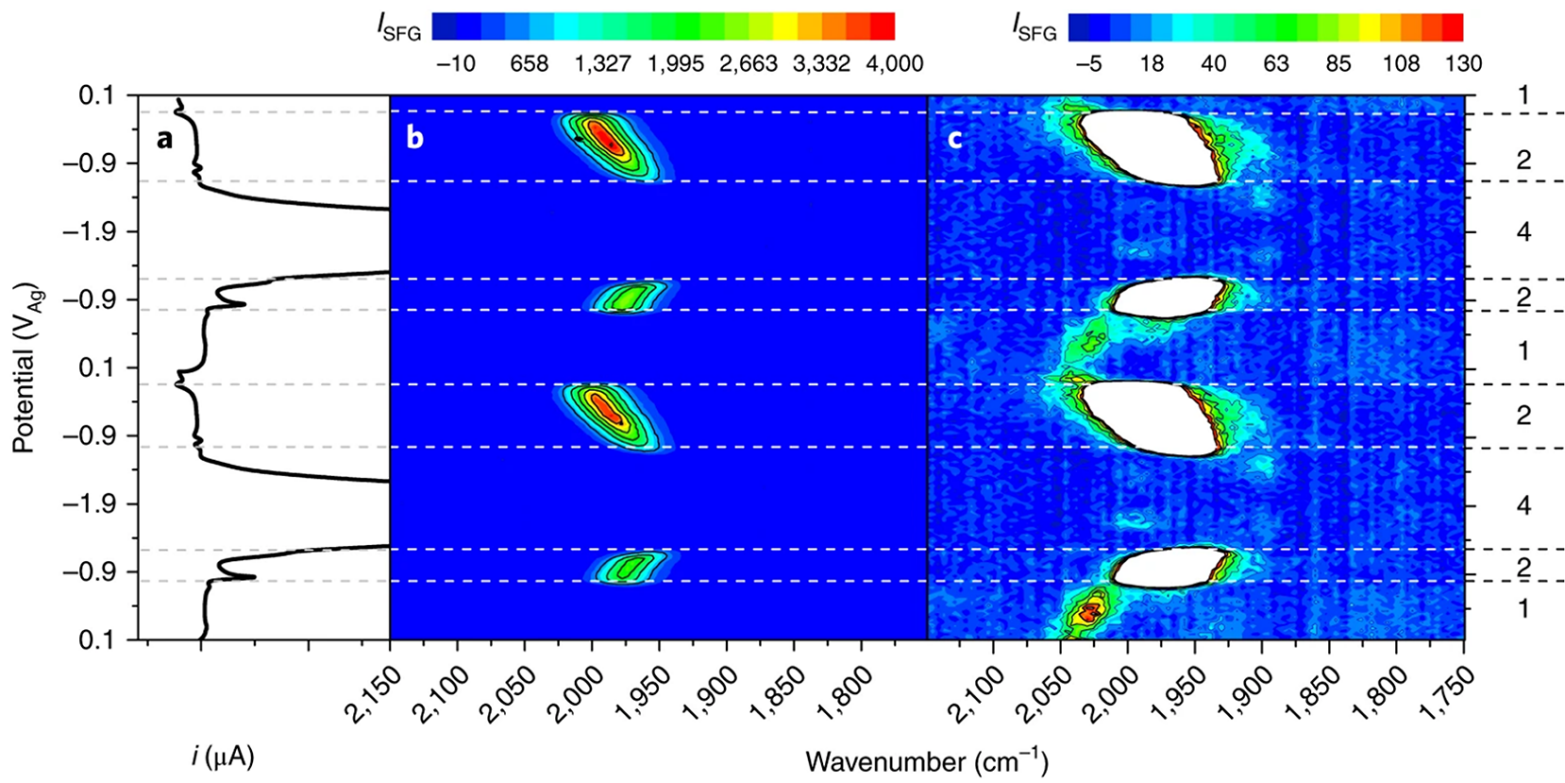
CV (a) of [Mn(bpy)(CO)_3_Br] in CH_3_CN in the presence of 1.5 M TFE under Ar and VSFG spectra (b, c) recorded in situ of the complex at the working electrode. Panel c is an expansion (*z*-axis, VSFG intensity) of panel b. The numbers on the right correspond to the spectral assignments. (1) [Mn(bpy)(CO)_3_(solv)]^+^; (2) [Mn_2_bpy_2_(CO)_6_]; (4) [Mn(bpy)(CO_3_)H]. Adapted from ref (3) with permission from Springer Nature.

Initially we have carried out experiments in acetonitrile with added Brønsted acids as the need for IR transmission through the electrolyte prevents the study of aqueous electrolytes. VSFG data recorded during a CV of [Mn(bpy)(CO)_3_Br] in CH_3_CN with TFE using a static Hg/Au amalgam electrode under an Ar atmosphere is shown in Figure 5.^3^ In the VSFG experiments, we focus on the metal carbonyl stretching modes as they act as excellent reporter groups on the state of the metal center and prior bulk SEC-FTIR studies^1,45−47^ provide a way to assign known species at the electrode surface. At open circuit, no ν(CO) bands were observed, but as the potential of the electrode was swept reductively from +0.1 V to −0.4 V (all potentials in the VSFG experiments in this section are versus a Ag pseudoreference electrode), a strong (∼2043 cm^–1^) ν(CO) band increased in intensity (Figure 5c). A second broad, much weaker band around ∼1960–1940 cm^–1^ could also be observed upon careful inspection of individual spectra (not shown here) with both bands assigned to [Mn(bpy)(CO)_3_(CH_3_CN)]^+^ (the solvent can displace the bromide ligand). The ν(CO) bands shifted in frequency with applied potential, giving a Stark shift of ∼35 cm^–1^ V^–1^, demonstrating that the vibrational spectra were occurring from [Mn(bpy)(CO)_3_(CH_3_CN)]^+^ experiencing a large electric field, which therefore must be at or near the electrode surface.

As expected from the past FTIR reports,^25,48^ the reduction of [Mn(bpy)(CO)_3_(CH_3_CN)]^+^ leads to formation of [Mn_2_(bpy)_2_(CO)_6_] (species 2, Figure 1b), and this in turn could be reduced at potentials negative of −0.9 V_Ag_. The *I*_SFG_ of the ∼1970 cm^–1^ resonant mode of [Mn_2_(bpy)_2_(CO)_6_] is very intense (Figure 5b) as the visible laser pulse is resonant with an electronic transition of this complex.^20^ We were unable to observe the anticipated active catalyst, [Mn(bpy)(CO)_3_]^−^, at the electrode surface. Instead, [Mn(bpy)(CO)_3_H] formed rapidly (Figure 5) in the absence of CO_2_. Using VSFG spectroscopy, we found, in both the absence of a deliberately added acid and the presence of a number of acids (methanol, TFE, phenol), at the electrode surface [Mn(bpy)(CO)_3_H] formation in the absence of CO_2_.^3,49^ It is clear that at the electrode surface [Mn(bpy)(CO)_3_H] formation occurs very rapidly even in the absence of a deliberately added Brønsted acid (trace water is likely present), and the cause of the high selectivity of this complex toward CO_2_ is not the lack of hydride formation. This is an interesting observation as FTIR spectroelectrochemistry had monitored the formation of [Mn(6,6′-dimesityl-2,2′-bipyridine)(CO)_3_]^−^ in the bulk electrolyte indicating its stability^50^ and DFT calculations^38,39^ predicted a ∼13–15 kcal mol^–1^ barrier to binding of H^+^ to [Mn(bpy)(CO)_3_]^−^. One possible rationale of the VSFG result may be the presence of the large electric field at the electrode interface, which can have a profound effect on the relative stability of the species,^51^ or due to preferential orientation/accumulation of protons at the electrode surface, with both situations highlighting the need to monitor the surface species.

**Figure 6:**
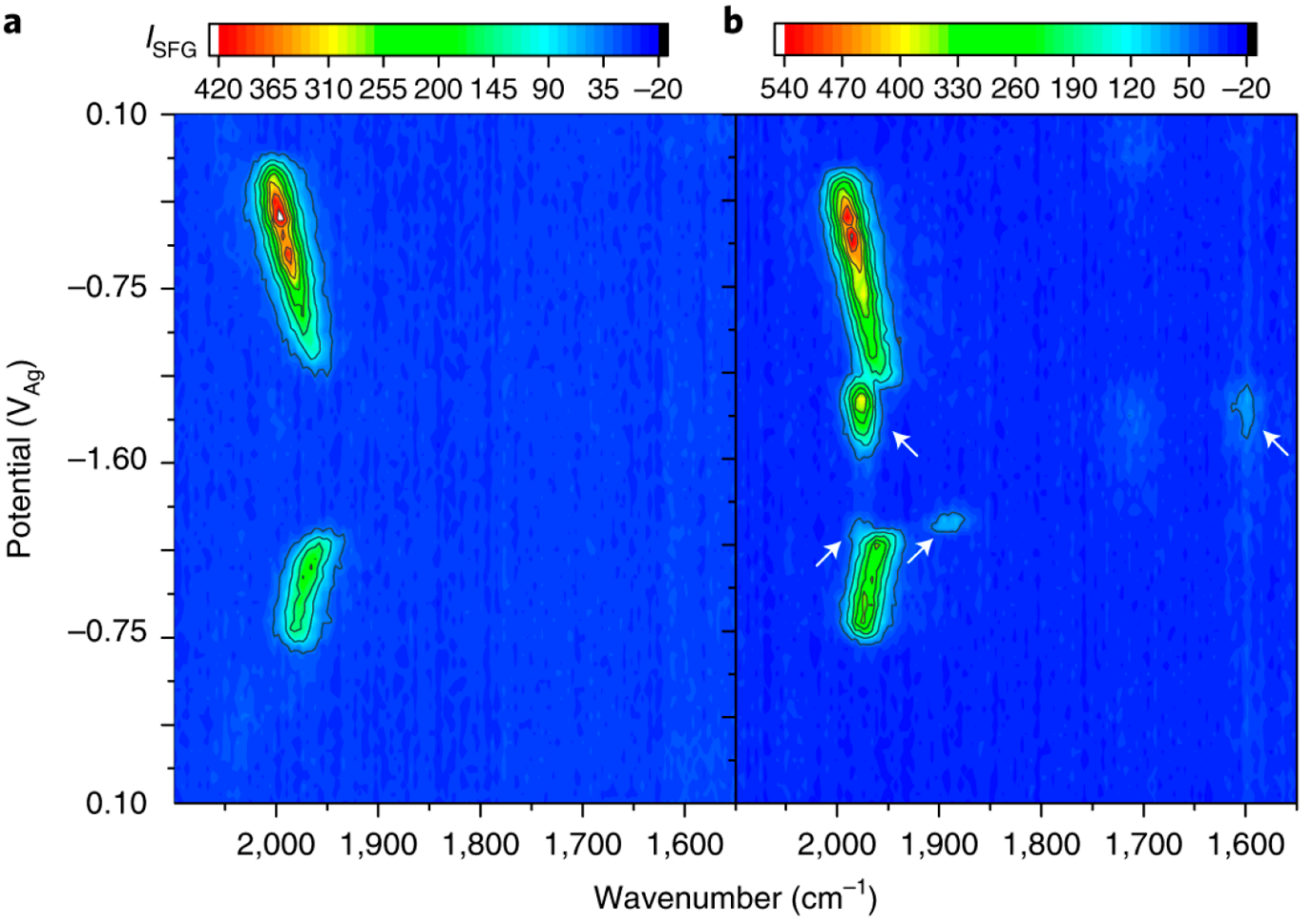
VSFG spectra of [Mn(bpy)(CO)_3_Br] in CH_3_CN in the presence of 1.5 M TFE under Ar (a) and CO_2_ (b). New CO_2_ reduction intermediates at the electrode surface are indicated with white arrows with the bands at ∼1976 and ∼1600 cm^–1^ due to [Mn(bpy)(CO)_4_]^+^ and a band at ∼1875 cm^–1^ possibly due to [Mn(bpy)(CO_2_H)]^−^. Reproduced from ref (3) with permission from Springer Nature.

Under CO_2_ with TFE present, in the potential region where CO_2_ reduction begins, [Mn(bpy)(CO)_3_H] is not detected; instead several new ν(CO) bands due to CO_2_ reduction intermediates appear, Figure 6.^3^ The intensity of the VSFG bands of the CO_2_ reduction intermediates was greatest with acids with low p*K*_a_ values. Under CO_2_ with the weakest acid studied (e.g., methanol), and when no acid was present, VSFG spectra showed only [Mn(bpy)(CO)_3_H] formation, confirming past predictions that CO_2_ binding to [Mn(bpy)(CO)_3_]^−^ is endergonic in the absence of a suitably strong acid.^39^ Instead in weak acids, catalysis is thought to occur via the formation of [Mn(bpy)(CO)_3_(CO_2_)]^2–^, which occurs only at potentials more negative than examined in our work.^39^

Past DFT and microkinetic simulations predicted that [Mn(bpy)(OCOH)] and [Mn(bpy(CO_2_H)]^−^ would be the main intermediates observed under CO_2_ when TFE and phenol were the acid sources at −1.7 and −2.0 V_SCE_, respectively.^37^ However, isotopic labeling experiments and DFT calculations of the Stark tuning rates of the ν(CO) modes of the CO_2_ intermediate ruled out assignment to [Mn(bpy)(OCOH)], and the VSFG bands at ∼1976 and 1600 cm^–1^ were assigned to [Mn(bpy)(CO)_4_]^+^, a later intermediate in the catalytic cycle of the “protonation first” pathway.^3^ The VSFG data did support the proposed potential dependent switching between a protonation first and reduction first pathway,^38,39^ with an additional band at ∼1875 cm^–1^ possibly being due to [Mn(bpy)(CO_2_H)]^−^. The availability of the lower-overpotential protonation first pathway catalysis had also been demonstrated to occur elsewhere in several studies with derivatives of the Mn complex,^52,53^ and its accessibility offers a further reason for the typically lower overpotentials and increased activity for CO_2_ reduction using this class of Mn complexes in the presence of stronger acids.^37,38^

With [Mn(bpy)(CO)_3_Br], a CO_2_ reduction current is also observed at potentials positive of [Mn_2_(bpy)_2_(CO)_6_] reduction, demonstrating the presence of an additional low-overpotential pathway to produce CO. The catalytic studies outlined in section 2 show that this “dimer pathway” (Figure 1b) also retains high selectivity toward CO production even in water. The mechanism of catalysis via the dimer was first studied through a combination of pulsed-EPR and UV/vis spectroscopy,^54^ where it was shown that in a 5% water/95% CH_3_CN solution CO_2_ purging led to loss of electrochemically generated dimer in the bulk electrolyte.^55^ Further UV/vis and FTIR studies of immobilized Mn catalysts also explored the reactivity of the dimer complex in the presence of water and showed that it was decreased within seconds of the electrolyte being exposed to CO_2_.^56,57^ However, the behavior of the dimer using different acid sources had not been previously studied in detail and VSFG spectroscopy also offered a way to analyze the possible role of surface specific species in the “dimer mechanism”.^4^

**Figure 7:**
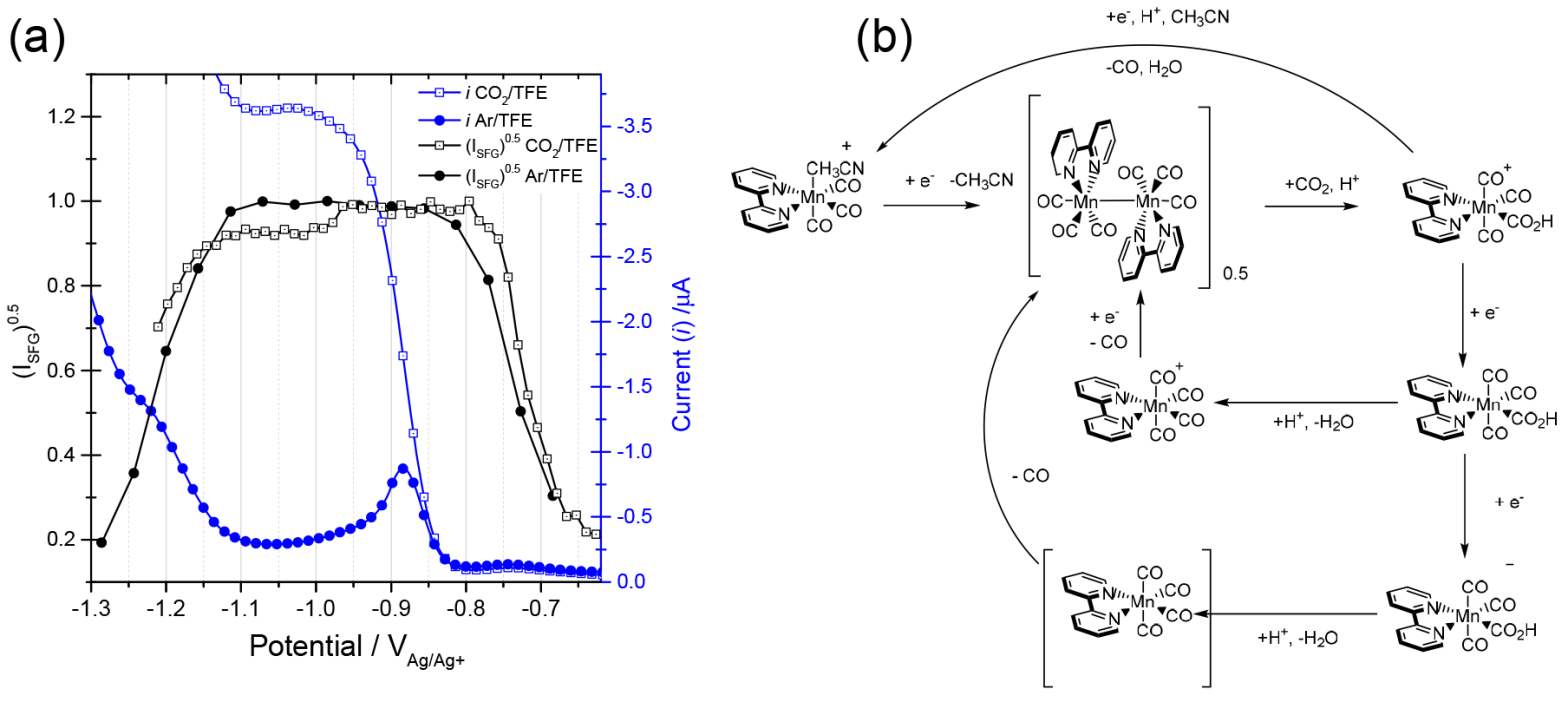
(a) Square root of VSFG intensity of a ν(CO) band of [Mn_2_(bpy)_2_(CO)_6_] under CO_2_ and Ar gives a measure of the concentration of this species at the electrode as the potential is changed. The current recorded during the experiment is in blue. (b) Analysis of VSFG data leads to a new proposed pathway (bottom) for CO production via the reduction of [Mn(bpy)(CO)_3_(CO_2_H)]. Reproduced from ref (4) with permission from the PCCP Owner Societies.

In a homodyne experiment, it can be approximated that VSFG signal intensities scale quadratically with the density of vibrational modes in the interface region.^41^ Therefore, a plot of the square root of the VSFG intensities versus electrode potential provides a semiquantitative measure of the surface/double layer concentration of the species. VSFG experiments looking at the behavior of [Mn_2_(bpy)_2_(CO)_6_] in CH_3_CN with a range of acids added showed that the dimer accumulated and reached a plateau concentration at −0.7 V_Ag_ (Figure 7a) in the presence of TFE.^4^ Under Ar, the surface population of [Mn_2_(bpy)_2_(CO)_6_] remained constant regardless of the acid used (TFE, phenol, no acid) until reduction occurred, and this led to the formation of [Mn(bpy)(CO)_3_H]. Identical behavior was observed under CO_2_ in the absence of an added acid, with [Mn_2_(bpy)_2_(CO)_6_] persisting at the electrode surface prior to [Mn(bpy)(CO)_3_H] formation occurring, indicating that CO_2_ is unable to interact with the dimer without a suitable Brønsted acid. In the presence of either TFE or phenol and CO_2_ a notable decrease in the surface concentration of [Mn_2_(bpy)_2_(CO)_6_] occurred, 130 mV positive of the reduction potential of [Mn_2_(bpy)_2_(CO)_6_]. The extent of decrease in the VSFG signal of [Mn_2_(bpy)_2_(CO)_6_] was greatest when the lowest p*K*_a_ acid (phenol) and CO_2_ were used indicating that CO_2_ interaction with the dimer to produce the previously^54^ detected *mer* Mn^II^–CO_2_H also occurs via a protonation-assisted CO_2_ binding mechanism. Furthermore, by analysis of the onset of the catalytic current and from knowledge of the electrochemical stability of previously proposed intermediates, a new alternative pathway for CO evolution following *mer*-Mn^II^–CO_2_H formation via the reduction of a *mer-*Mn(bpy)(CO)_3_(CO_2_H) intermediate occurs prior to protonation and H_2_O loss (Figure 7b), different from those previously put forward by Deronzier and Grills.^22,54^

## 4 Conclusions and Outlook

VSFG spectroscopy can follow the Mn electrocatalyst for CO_2_ reduction while at the electrode surface, and our results complement the theoretical and other spectroscopic studies in the literature to provide important insights into the remarkable selectivity of this class of catalysts toward CO_2_ reduction. The low levels of H_2_ production are not due to a lack of H^+^ binding when the active [Mn(bpy)(CO)_3_]^−^ catalyst is generated as previously postulated, as [Mn(bpy)(CO)_3_H] forms rapidly at the electrode surface in the absence of CO_2_. Instead selectivity toward CO_2_ in all catalytic pathways (“dimer pathway” species 2 and 11; “protonation first” species 3, 5, 6, 8, and 9, and “reduction first” species 3, 5, 6, 7, and 9, Figure 1b) arises by an unusual acid-promoted CO_2_ binding mechanism, where acids with a lower p*K*_a_ actually lead to higher concentrations of CO_2_ reduction intermediates. We believe that such an effect has been rarely reported within the large variety of known homogeneous CO_2_ reduction catalysts. To date, our studies have focused on one particular class of catalysts and not in aqueous solvent, which may complicate direct comparisons, but we encourage future works to explore if similar proton-assisted CO_2_ binding mechanisms are in wider operation among CO_2_ reduction catalysts.

Recent technoeconomic analyses highlight the need to understand and discover new electrocatalysts that can reduce CO_2_ selectively in water, in particular at low pH.^27^ There are relatively few studies to date on the use of this class of catalysts in water, but from the emerging literature, it does appear that the proton-assisted CO_2_ binding mechanisms seen in aprotic solvents may be facilitating the measured high levels of selectivity in aqueous electrolytes as well. While the long-term stability of these Mn catalysts is uncertain, especially under high current densities, initial catalysis studies are promising, and these Mn complexes serve as valuable models for future development of proton/acid tolerant CO_2_ reduction catalysts.
